# Optimal Dietary Lipid Requirement for Juvenile Leopard Coral Grouper (*Plectropomus leopardus*)

**DOI:** 10.1155/anu/9438737

**Published:** 2026-05-04

**Authors:** Xiangqin Lin, Yixiong Cao, Xiaoxue Meng, Shiwei Xie, Shuyan Chi, Shuang Zhang, Beiping Tan, Junming Deng

**Affiliations:** ^1^ College of Fisheries, Guangdong Ocean University, Zhanjiang, 524088, China, gdou.edu.cn

**Keywords:** antioxidant status, dietary lipid, growth performance, lipid metabolism, *Plectropomus leopardus*

## Abstract

Leopard coral grouper (*Plectropomus leopardus*) is a high‐value marine species increasingly produced under intensive farming conditions; however, its dietary lipid requirement remains poorly defined, hindering the development of cost‐effective formulated feeds. This study aimed to determine the optimal dietary lipid level for juvenile *P. leopardus* by assessing growth performance, hepatic lipid metabolism, and antioxidant capacity in response to graded lipid inclusion. Juveniles (initial body weight [IBW]: 13.94 ± 0.07 g) were fed six isonitrogenous diets (53% crude protein) containing 6%, 8%, 10%, 12%, 14%, or 16% lipid for 9 weeks (three replicate tanks per diet; 27 fish per tank). Weight gain (WG) and specific growth rate (SGR) increased with dietary lipid up to 10% and then declined. Intestinal lipase activity increased with dietary lipid level. Dietary lipid at 8%–12% improved hepatic lipid metabolic balance, as indicated by reduced activities of lipogenic enzymes (malate dehydrogenase [MDH], glucose‐6‐phosphate dehydrogenase [G6PD], and fatty acid synthase [FAS]) and increased lipoprotein lipase (LPL) activity (*p* < 0.05). Dietary lipid at 8%–12% also enhanced antioxidant defenses, reflected by higher glutathione peroxidase (GPx) and superoxide dismutase (SOD) activities in serum, liver, and hindgut, without an increase in malondialdehyde (MDA). In contrast, high‐lipid diets (14%–16%) induced hepatic lipid metabolic imbalance followed by pronounced hepatic lipid deposition (histological staining and increased hepatic crude lipid; *p* < 0.05), elevated serum alanine aminotransferase (ALT) and aspartate aminotransferase (AST) activities, and significantly increased MDA levels in serum, liver, and hindgut (*p* < 0.05), while antioxidant enzyme activities tended to decrease, indicating heightened oxidative stress. Overall, growth performance was highest at ~10% dietary lipid under the present experimental conditions, and quadratic regression analysis suggested an approximate optimum near 9.3%. Moderate dietary lipid (8%–12%) promoted growth by improving hepatic lipid metabolism and antioxidant capacity, whereas excessive lipid inclusion (≥14%) induced excessive hepatic lipid accumulation and oxidative damage in *P. leopardus*.

## 1. Introduction

Leopard coral grouper (*Plectropomus leopardus*) is a warm‐water, reef‐associated marine fish distributed from the western Pacific to East Africa and the Red Sea [1]. Owing to its vivid coloration, this species is valued both as an ornamental fish and as a premium product in live fish markets across Southeast Asia, including China, Indonesia, and Japan [2]. In recent decades, overfishing, combined with increasing market demand, has led to a marked decline in wild stocks [3, 4]. Advances in hatchery and grow‐out technologies have improved fry availability and promoted a shift toward intensive farming [5]. However, intensive production remains constrained by high costs, largely due to the lack of species‐specific formulated feeds. At present, *P. leopardus* is still largely fed raw or chilled baitfish/trash fish, which do not consistently meet its nutritional requirements [6, 7]. Therefore, defining the nutritional requirements of *P. leopardus* and developing high‐quality formulated diets are essential for improving production efficiency and supporting the sustainable development of this industry. Recent studies have begun to establish a nutritional framework for juvenile *P. leopardus*, including evaluations of dietary protein [8], requirement studies for lysine [7] and phospholipids [9], and functional assessments such as tryptophan supplementation [10]. However, quantitative evidence to define the optimal dietary lipid level for juvenile *P. leopardus* remains limited.

Dietary lipid is a major nutrient for fish growth and exerts diverse physiological and metabolic effects [11]. In addition to serving as a major energy source and supplying essential fatty acids, lipids facilitate the absorption and transport of fat‐soluble nutrients [12]. Lipids are key structural components of cell membranes (e.g., phospholipids and sterols), thereby supporting membrane integrity and cellular function [13]. After digestion and absorption by intestinal lipases, dietary lipids are re‐esterified and transported to the liver mainly as triglyceride (TAG)‐rich lipoproteins. In the liver, lipid homeostasis is maintained through coordinated lipogenic and lipolytic/oxidative pathways [14]. When these pathways are properly balanced, lipid peroxidation and reactive oxygen species (ROS) generation are limited, as reflected by enhanced antioxidant defenses and reduced oxidative damage markers [15]. For example, in turbot (*Scophthalmus maximus*), increasing dietary lipid improved growth, increased hepatic superoxide dismutase (SOD) activity without elevating malondialdehyde (MDA), and significantly altered the expression of lipid metabolism–related genes (e.g., lipoprotein lipase [LPL]) [16]. These growth benefits are often attributed, at least in part, to a protein‐sparing effect, whereby lipid supplies energy and conserves dietary protein for tissue accretion [17]. In contrast, excessive dietary lipid can disrupt the balance between hepatic lipogenesis and lipolysis, resulting in abnormal lipid deposition [18]. Lipid overload can also increase ROS production, potentially via enhanced β‐oxidation and mitochondrial respiration, thereby promoting lipid peroxidation (e.g., elevated MDA) and weakening antioxidant defenses. These changes may compromise hepatocellular integrity and subsequently impair liver function (as indicated by increased serum alanine aminotransferase [ALT] and aspartate aminotransferase [AST] activities), which may ultimately contribute to growth suppression [19]. Similar responses have been reported in largemouth bass (*Micropterus salmoides*) fed high‐lipid diets [20]. Overall, dietary lipid is indispensable for juvenile fish growth; however, inclusion levels must be carefully balanced to avoid metabolic disturbance and compromised health.

Therefore, we assessed how graded dietary lipid levels influence growth performance, antioxidant capacity, and hepatic lipid metabolism in juvenile *P. leopardus*, and we integrated growth responses with key metabolic and health‐related endpoints to identify an inclusion level that supports performance without inducing hepatic lipid deposition or oxidative damage. This study fills a key knowledge gap in the lipid nutrition of *P. leopardus* and provides a scientific basis for developing precision‐formulated diets for its intensive aquaculture.

## 2. Materials and Methods

### 2.1. Ethics Statement

All procedures were approved by the Animal Research and Ethics Committee of Guangdong Ocean University (IACUC‐GDOU‐10/2025‐A0074) and complied with the Chinese Guide for the Care and Use of Laboratory Animals (GB/T 35892‐2018).

### 2.2. Experimental Diets

Six isonitrogenous diets (53% crude protein) were formulated to contain 6%, 8%, 10%, 12%, 14%, or 16% lipid, with fish oil and soybean oil (1:1, w/w) used as the lipid source (Table 1). The fatty acid profiles of the diets are provided (Table 2). Diets were prepared following standard laboratory procedures, as described previously [7]. All dry ingredients were ground and passed through a 40‐mesh sieve to ensure a uniform particle size. The dry mixture was homogenized using a laboratory mixer (M‐256; South China University of Technology, Guangzhou, China). Thereafter, the oil blend and water were added sequentially while mixing. The resulting dough was pelleted using a twin‐screw extruder (F‐26; South China University of Technology, Guangzhou, China). After extrusion, pellets were air‐dried at 16°C for 48 h to a final moisture content of ~10% and then stored at −20°C until use. Diet formulation targets were set to meet the nutritional requirements of juvenile *P. leopardus* and were calculated based on the analyzed nutrient composition of each ingredient. In addition, representative diet samples were analyzed to verify proximate composition.

**Table 1:** Formulation and proximate composition (% dry matter) of the experimental diets fed to leopard coral grouper (*Plectropomus leopardus*).

Ingredients	Dietary lipid level (%)					
	6	8	10	12	14	16
White fish meal^1^	50.00	50.00	50.00	50.00	50.00	50.00
Soy protein concentrate^1^	12.00	12.00	12.00	12.00	12.00	12.00
Casein^2^	6.00	6.00	6.00	6.00	6.00	6.00
Gelatin^2^	1.50	1.50	1.50	1.50	1.50	1.50
Wheat flour^1^	15.50	15.50	15.50	15.50	15.50	15.50
Soybean lecithin^1^	2.00	2.00	2.00	2.00	2.00	2.00
Fish oil^1^	0.00	1.00	2.00	3.00	4.00	5.00
Soybean oil^1^	0.00	1.00	2.00	3.00	4.00	5.00
Microcrystalline cellulose^3^	10.00	8.00	6.00	4.00	2.00	0.00
Vitamin premix^4^	0.50	0.50	0.50	0.50	0.50	0.50
Mineral premix^4^	1.00	1.00	1.00	1.00	1.00	1.00
Others^5^	1.50	1.50	1.50	1.50	1.50	1.50
Proximate composition						
Dry matter (DM, %)	90.24	91.02	90.92	90.82	90.76	91.58
Crude protein (% DM)	54.54	53.24	52.95	53.33	53.18	53.06
Crude lipid (% DM)	5.92	7.46	10.04	11.60	13.48	15.47
Ash (% DM)	13.07	12.91	12.94	12.97	13.15	13.41

^1^Dietary ingredients were provided by Guangdong Yuehai Feed Group Co., Ltd. (Zhanjiang, China).

^2^Casein and gelatin were provided by Zhengzhou Yuhe Food Additives Co., Ltd. (Zhengzhou, China).

^3^Microcrystalline cellulose was provided by Huzhou Linghu Xinwang Chemical Co., Ltd. (Huzhou, China).

^4^Vitamin and mineral premixes were provided by Qingdao Master Bio‐Tech. Co., Ltd. (Qingdao, China).

^5^Others included 1.0% Ca(H_2_PO_4_)_2_, 0.4% choline chloride, 0.05% yttrium oxide, 0.03% vitamin C, and 0.02% ethoxyquin.

**Table 2:** Fatty acid composition of experimental diets (% of total fatty acid).

Fatty acids	Dietary lipid levels (%)					
	6	8	10	12	14	16
14:0	3.09	2.15	1.74	1.47	1.29	1.20
16:0	18.47	16.20	15.04	14.37	14.04	13.08
18:0	5.38	5.30	5.23	5.23	5.22	5.21
20:0	0.40	0.45	0.47	0.49	0.49	0.50
∑SFA	27.34	24.10	22.48	21.56	21.04	20.71
16:1n‐7	3.59	2.83	2.51	2.31	2.16	2.09
18:1n‐9	21.70	23.02	23.30	23.69	23.90	24.01
20:1n‐9	2.36	2.18	2.10	2.09	2.03	2.01
∑MUFA	27.65	28.03	27.91	28.09	28.09	28.11
18:2n‐6	22.01	25.16	26.61	27.27	28.22	28.39
20:2n‐6	0.23	0.23	0.23	0.25	0.24	0.25
20:4n‐6	0.62	0.73	0.80	0.85	0.86	0.87
∑n‐6 PUFA	22.86	26.12	27.64	28.37	29.32	29.51
18:3n‐3	2.82	3.25	3.53	3.61	3.72	3.76
20:5n‐3	9.65	9.20	9.28	9.16	8.99	8.99
22:6n‐3	7.13	6.82	6.79	6.68	6.59	6.57
∑n‐3 PUFA	19.60	19.27	19.60	19.45	19.30	19.32
n‐3/n‐6 PUFA	0.86	0.74	0.71	0.69	0.66	0.65
DHA/EPA	0.74	0.74	0.73	0.73	0.73	0.73

*Note*: DHA/EPA: 22:6n‐3/20:5n‐3.

Abbreviations: SFAs, saturated fatty acids; MUFAs, monounsaturated fatty acids; n‐3 PUFA, n‐3 polyunsaturated fatty acids; n‐6 PUFA, n‐6 polyunsaturated fatty acids.

### 2.3. Fish and Husbandry Management

Healthy juvenile leopard coral groupers were acquired from the Hengxing Breeding Base (Zhanjiang, China). After transport to the experimental facility, fish were acclimated for 2 weeks and fed a commercial hybrid grouper diet (52% crude protein and 8 % crude lipid). After acclimation, juveniles of uniform size (initial body weight [IBW]: 13.90 g) were randomly assigned to 18 fiberglass tanks (0.7 m diameter, 0.8 m height, and volume: 0.3 m^3^), with 27 fish per tank. Six dietary treatments were tested, with three replicate tanks per treatment (81 fish per treatment and 486 fish in total). The 9‐week feeding trial was conducted in a flow‐through system under a natural photoperiod. Fish were hand‐fed to apparent satiation twice daily (08:00 and 17:00). Water quality parameters were monitored daily: temperature, 30.0 ± 1.0°C; pH, 7.85 ± 0.15; salinity, 30.0 ± 1.0‰; total ammonia nitrogen, <0.20 mg/L; nitrite nitrogen, <0.05 mg/L; and dissolved oxygen, >5.0 mg/L.

### 2.4. Sample Collection

At the end of the feeding trial, fish were fasted for 24 h before sampling. All fish in each tank were anesthetized with eugenol (1:10,000), counted, and individually weighed. Body weight was recorded for each individual. Three fish per tank were randomly selected, frozen at −20°C, and used for whole‐body proximate composition analysis. Blood was collected from the caudal vein of three fish per tank, kept at 4°C for 4 h, and centrifuged at 3000 × g for 10 min at 4°C. The serum was then collected and stored at −80°C for biochemical assays.

After blood sampling, the liver and viscera were excised and weighed to calculate somatic indices. Livers from five fish per tank were divided into three portions: One was fixed in 4% formaldehyde for histological analysis, one was snap‐frozen in liquid nitrogen and stored at −80°C for enzyme activity assays, and the remaining portion was stored at −20°C. Foregut and hindgut segments were collected from three fish per tank. The foregut was used for digestive enzyme assays, whereas the hindgut was used for antioxidant analyses. Dorsal muscle samples were collected for proximate composition and fatty acid analyses.

### 2.5. Sample Determination

#### 2.5.1. Proximate Composition

Proximate composition of the whole body, liver, and dorsal muscle was determined according to AOAC [21] International methods. Moisture content was determined by oven‐drying at 105°C to constant weight. Crude protein was determined using the Kjeldahl method. Crude lipid was extracted with diethyl ether using a Soxhlet apparatus, and ash content was determined after incineration at 550°C for 16 h.

#### 2.5.2. Fatty Acid Analysis of Diet and Muscle

Experimental diets and dorsal muscle samples were freeze‐dried under vacuum for 48 h and then ground to a fine powder. Total lipids were extracted using chloroform–methanol (2:1, v/v) following the method of Folch et al. [22]. Briefly, lipid extracts were saponified with 0.5 M methanolic KOH at 60°C for 15 min and then methylated with methanol─HCl (3 M) at 60°C for 60 min to produce fatty acid methyl esters (FAMEs). After cooling, FAMEs were extracted with n‐hexane, and the organic phase was dried over anhydrous Na_2_SO_4_ prior to gas chromatography (GC) analysis. Fatty acid profiles were determined using a gas chromatograph (Hitachi 263‐30; Tokyo, Japan) equipped with a flame ionization detector (FID) and a CP‐Sil 88 capillary column for FAME analysis. The oven temperature was programmed from 120°C (held for 2 min) to 175°C at 10°C/min (held for 10 min) and then to 220°C at 5°C/min (held for 15 min). Fatty acids were identified by comparing retention times with those of a commercial 37‐component FAME standard mixture (Supelco, CRM47885, Sigma–Aldrich, St. Louis, MO, USA). Results are expressed as a percentage of total identified fatty acids (% of total FA).

#### 2.5.3. Intestinal Digestive Enzyme Activities

Foregut samples were homogenized in ice‐cold physiological saline at a 1:4 (w/v) ratio, following the method of Deng et al. [23] with minor modifications. The homogenates were centrifuged at 10,000 × g for 20 min at 4°C, and the supernatants were collected. The supernatants were further diluted with physiological saline to the required dilution (appropriate for each assay) and used as crude enzyme extracts for subsequent enzyme activity measurements.

The supernatants were used as crude enzyme extracts for subsequent assays. Trypsin (ML064285), lipase (ML093059), and amylase (ML036449) activities in the foregut were measured using commercial assay kits (Shanghai Enzyme‐linked Biotechnology Co., Ltd., Shanghai, China). Enzyme activities were expressed as specific activity (U mg^−1^ protein). Protein concentration was determined using the Bradford [24] method.

#### 2.5.4. Serum Biochemical Parameters

Commercial assay kits (Nanjing Jiancheng Bioengineering Institute, Nanjing, China) were used to determine four serum lipid indices and the activities of two liver function–related enzymes (ALT and AST). Serum total cholesterol (TC), TAGs, high‐density lipoprotein cholesterol (HDL‐C), and low‐density lipoprotein cholesterol (LDL‐C) were measured using kits A111‐1‐1, A110‐1‐1, A112‐1‐1, and A113‐1‐1, respectively. Serum ALT and AST activities were measured using kits C009‐2‐1 and C010‐2‐1, respectively.

#### 2.5.5. Hepatic Lipid–Metabolizing Enzyme Activities

The activities of fatty acid synthase (FAS), malate dehydrogenase (MDH), glucose‐6‐phosphate dehydrogenase (G6PD), LPL, hormone‐sensitive lipase (HSL), and carnitine palmitoyltransferase‐1 (CPT‐1) were quantified using commercial ELISA kits (Shanghai Enzyme‐linked Biotechnology Co., Ltd., Shanghai, China) with Cat. Nos. ML076654, ML092980, ML095182, ML016889, ML026141, and ML076617, respectively.

#### 2.5.6. Liver Histology

Fixed liver samples were processed using two histological protocols. For paraffin histology, samples were fixed in 4% paraformaldehyde for 24 h, dehydrated via a graded ethanol series (70%, 80%, 90%, 95%, and 100%), cleared in xylene, embedded in paraffin, and sectioned at 5 μm using a rotary microtome. Sections were stained with hematoxylin and eosin (H&E) following standard procedures, including deparaffinization, rehydration, staining, dehydration, clearing, and mounting. For lipid visualization, samples were embedded in OCT compound, cryosectioned at 10 μm using a cryostat, and stained with Oil Red O to visualize neutral lipid droplets. Sections were briefly differentiated in 70% ethanol, rinsed, and mounted using an aqueous mounting medium. All sections were examined and imaged using an inverted microscope (Eclipse Ti‐E; Nikon, Japan) under identical imaging settings; representative images were captured at 400× magnification.

#### 2.5.7. Antioxidant‐Related Parameters in Serum, Hindgut, and Liver

Hindgut and liver tissues were processed as described by Deng et al. [23] with minor modifications. Antioxidant‐related parameters in serum, hindgut, and liver were measured using commercial assay kits (Nanjing Jiancheng Bioengineering Institute, Nanjing, China). The parameters included SOD (A001‐3‐1), glutathione peroxidase (GPx; A005‐1‐1), total antioxidant capacity (TAC; A015‐2‐1), and MDA (A003‐1‐1). For liver and hindgut tissues, results were expressed as activity or content per gram of protein. Protein concentration was determined using the Bradford [24] method.

### 2.6. Calculation and Statistical Analysis

Weight gainWG=final body weightg–initial body weightg/initial body weightg,

Specific growth rateSGR,%/d=lnfinal body weight– lninitial body weight/feeding days×100,

Feed intakeFI,%/d= feed consumption/initial body weightg+final body weightg/2/feeding days×100,

Feed conversion ratioFCR= dry feed fed/final body weightg–initial body weightg,

Protein efficiency ratioPER=final body weightg–initial body weightg/dietary protein intakeg,

Hepatosomatic indexHSI,%=100×hepatic weightg/body weightg,

Viscerosomatic indexVSI,%=100×visceral weightg/body weightg.

Data are presented as mean ± SEM. Statistical analyses were performed using SPSS 17.0 (SPSS Inc., Chicago, IL, USA). Data were tested for normality (Kolmogorov–Smirnov test) and homogeneity of variances (Levene’s test) before one‐way ANOVA. Differences among dietary treatments were evaluated by one‐way ANOVA, followed by Duncan’s multiple range test for post hoc comparisons (*p* < 0.05).

The optimal dietary lipid level for *P. leopardus* was estimated by quadratic regression using weight gain (WG) as the response variable. Quadratic regression was selected because it is widely used to estimate nutrient requirements and can capture nonlinear responses of growth to graded nutrient levels [25–27]. Model fit was evaluated using *R* ^2^ and the significance of the regression. The 95% confidence interval (CI) for the estimated optimal value was calculated to assess estimation precision. Allometric analysis was conducted using the equation [28]:

logy= a+ b logx,

where *y* is the whole‐body moisture, protein, lipid, or ash content (g); and *x* is the corresponding fish weight (g).

## 3. Results

### 3.1. Growth Performance and Body Index

**Figure 1:**
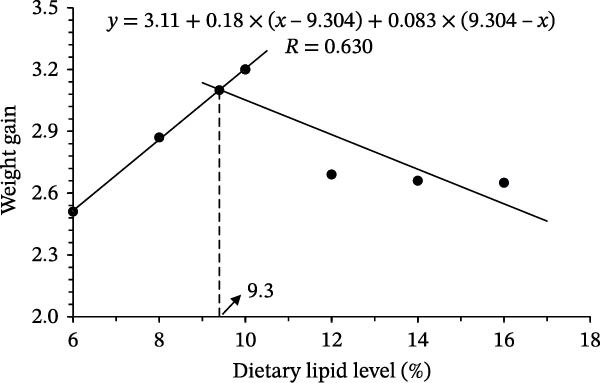
Dietary lipid requirement of juvenile leopard coral grouper (*Plectropomus leopardus*) based on weight gain.

Final body weight (FBW), WG, and specific growth rate (SGR) increased with dietary lipid up to 10% but declined at higher lipid levels (Table 3). Fish fed 10% lipid had significantly higher FBW, WG, and SGR than those fed 6%, 12%, 14%, or 16% lipid (*p* < 0.05). Protein efficiency ratio (PER) increased with dietary lipid up to 10% and remained relatively stable at higher lipid levels. Feed intake (FI) was significantly lower in fish fed 8%–16% lipid than in fish fed 6% lipid (*p* < 0.05). CF increased up to 10% lipid and declined thereafter. In contrast, viscerosomatic index (VSI) and hepatosomatic index (HSI) increased linearly with dietary lipid level. Quadratic regression of WG suggested that the dietary lipid level was ~9.3% (Figure 1), with a 95% CI of 8.62%–9.98%, although the relatively low *R* ^2^ value (0.236) indicates limited model strength. Quadratic regression of additional growth indices suggested similar estimated optima: 8.97% for FBW (95% CI: 8.21%–9.73%), 9.15% for SGR (95% CI: 8.45%–9.85%), and 9.42% for PER (95% CI: 8.76%–10.08%).

**Table 3:** Effect of dietary lipid level on growth performance and body indices of leopard coral grouper (*Plectropomus leopardus*).

Parameter	Dietary lipid levels (%)						Regression analysis		
	6	8	10	12	14	16	Regression equation	*R* ^2^	*p*‐Value
IBW (g)	13.99 ± 0.03	13.99 ± 0.01	13.91 ± 0.03	13.90 ± 0.04	13.96 ± 0.00	13.93 ± 0.05			
FBW (g)	49.02 ± 0.92^a^	54.15 ± 4.26^ab^	58.41 ± 0.18^b^	51.22 ± 0.26^a^	51.14 ± 1.32^a^	50.88 ± 1.96^a^	*y* = −0.198*x* ^2^ + 4.251*x* + 31.937	0.229	0.143
WG	2.51 ± 0.07^a^	2.87 ± 0.30^ab^	3.20 ± 0.02^b^	2.69 ± 0.03^a^	2.66 ± 0.10^a^	2.65 ± 0.15^a^	*y* = −0.0146*x* ^2^ + 0.317*x* + 1.223	0.236	0.133
SGR (%/d)	2.05 ± 0.04^a^	2.21 ± 0.13^ab^	2.35 ± 0.01^b^	2.14 ± 0.01^a^	2.13 ± 0.04^a^	2.12 ± 0.07^a^	*y* = −0.006*x* ^2^ + 0.133*x* + 1.484	0.278	0.087
FI (%/d)	2.93 ± 0.03^c^	2.31 ± 0.14^b^	2.13 ± 0.03^ab^	2.09 ± 0.05^ab^	2.12 ± 0.06^ab^	1.93 ± 0.11^a^	*y* = 0.013*x* ^2^–0.372*x* + 4.587	0.791	<0.001
FCR	1.26 ± 0.03^b^	1.20 ± 0.03^b^	1.07 ± 0.01^a^	1.11 ± 0.02^a^	1.11 ± 0.02^a^	1.06 ± 0.03^a^	*y* = 0.003*x* ^2^–0.079*x* + 1.637	0.666	<0.001
PER	1.26 ± 0.03^a^	1.69 ± 0.08^b^	1.95 ± 0.01^c^	1.83 ± 0.06^bc^	1.84 ± 0.03^bc^	2.00 ± 0.08^c^	*y* = –0.010*x* ^2^ + 0.287*x*–0.013	0.747	<0.001
CF (g/cm^3^)	1.94 ± 0.11^a^	2.19 ± 0.01^b^	2.48 ± 0.03^c^	2.21 ± 0.06^b^	2.23 ± 0.05^b^	2.25 ± 0.03^b^	*y* = −0.010*x* ^2^ + 0.238*x* + 0.912	0.461	0.010
HSI (%)	0.98 ± 0.05^a^	1.09 ± 0.11^ab^	1.15 ± 0.03^ab^	1.19 ± 0.02^bc^	1.35 ± 0.01^c^	1.55 ± 0.03^d^	*y* = 0.052*x* + 0.645	0.793	<0.001
VSI (%)	3.63 ± 0.08^a^	5.15 ± 0.19^b^	5.66 ± 0.33^bc^	5.61 ± 0.33^bc^	6.10 ± 0.10^c^	6.96 ± 0.22^d^	*y* = 0.278*x* + 2.458	0.796	<0.001

*Note*: Values are mean ± SEM (*n* = 3 tanks per treatment; one pooled sample per tank). Means within the same row that do not share a common superscript letter are significantly different (*p*  < 0.05). IBW, initial body weight (g); FBW, final body weight (g); weight gain (WG) = (final body weight (g) – initial body weight (g))/initial body weight (g); specific growth rate (SGR, %/d) = (ln (final body weight) – ln (initial body weight))/feeding days × 100; feed intake (FI, %/d) = feed consumption/[(initial body weight (g) + final body weight (g))/2]/feeding days × 100; feed conversion ratio (FCR) = dry feed fed/(final body weight (g) – initial body weight (g)); protein efficiency ratio (PER) = (final body weight (g) – initial body weight (g))/dietary protein intake (g); hepatosomatic index (HSI, %) = 100 × hepatic weight (g)/body weight (g); viscerosomatic index (VSI, %) = 100 × viscerosomatic weight (g)/body weight (g).

### 3.2. Intestinal Digestive Enzyme Activities

Intestinal lipase activity increased linearly with dietary lipid level and was significantly higher in the 14% and 16% lipid groups than in the 6% and 8% groups (Table 4). In contrast, intestinal amylase activity decreased as dietary lipid level increased, and fish fed 16% lipid showed significantly lower amylase activity than all the other groups. Trypsin activity did not differ significantly among dietary treatments (*p* > 0.05).

**Table 4:** Effect of dietary lipid level on digestive enzyme activities in the foregut of leopard coral grouper (*Plectropomus leopardus*).

Parameter	Dietary lipid level (%)						Regression analysis		
	6	8	10	12	14	16	Regression equation	*R* ^2^	*p*‐Value
Trypsin (KU/mg protein)	5.05 ± 0.21	5.03 ± 0.35	5.09 ± 0.32	4.94 ± 0.13	5.72 ± 0.12	5.83 ± 0.35	—	—	—
Lipase (U/mg protein)	1.49 ± 0.02^a^	1.51 ± 0.09^a^	1.67 ± 0.06^ab^	1.59 ± 0.04^ab^	1.94 ± 0.16^b^	1.96 ± 0.19^b^	*y* = 0.052*x* + 1.125	0.359	0.001
Amylase (U/mg protein)	0.29 ± 0.01^b^	0.23 ± 0.01^b^	0.23 ± 0.04^b^	0.23 ± 0.01^b^	0.24 ± 0.01^b^	0.16 ± 0.03^a^	*y* = –0.0004*x* ^2^–0.0015*x* + 0.288	0.347	0.017

*Note*: Values are mean ± SEM (*n* = 3 tanks per treatment; one pooled sample per tank). Means within the same row that do not share a common superscript letter are significantly different (*p*  < 0.05).

### 3.3. Serum Biochemical Parameters

Serum TC and TAG concentrations increased linearly with dietary lipid level (Table 5). Serum HDL‐C and LDL‐C increased with dietary lipid up to 12% and 10%, respectively, and then remained relatively stable at higher lipid levels. The HDL‐C/LDL‐C ratio exhibited a quadratic response, peaking in the 8% lipid group. Serum AST and ALT activities increased with dietary lipid level and were significantly higher in the 14% and 16% lipid groups than in the 6% lipid group (*p* < 0.05).

**Table 5:** Effect of dietary lipid level on serum lipoprotein level and transaminase activity in leopard coral grouper (*Plectropomus leopardus*).

	Dietary lipid levels (%)						Regression analysis		
Parameter	6	8	10	12	14	16	Regression equation	*R* ^2^	*p*‐Value
TC (mmol/L)	2.83 *±* 0.28^a^	4.88 ± 0.42^b^	5.00 ± 0.39^b^	5.26 ± 0.48^bc^	6.26 ± 0.21^cd^	7.26 ± 0.54^d^	*y* = 0.371*x* + 1.197	0.693	<0.001
TAG (mmol/L)	0.27 ± 0.05^a^	0.45 ± 0.07^ab^	0.47 ± 0.10^ab^	0.88 ± 0.22^bc^	0.90 ± 0.08^bc^	1.21 ± 0.28^c^	*y* = 0.093*x*–0.323	0.637	<0.001
HDL‐C (mmol/L)	1.67 ± 0.05^a^	2.15 ± 0.14^a^	2.35 ± 0.14^ab^	2.93 ± 0.37^b^	2.89 ± 0.16^b^	2.99 ± 0.19^b^	*y* = –0.013*x* ^2^ + 0.422*x* + 0.423	0.517	<0.001
LDL‐C (mmol/L)	0.34 ± 0.04^a^	0.40 ± 0.02^a^	0.77 ± 0.11^b^	0.71 ± 0.05^b^	0.74 ± 0.04^b^	0.79 ± 0.04^b^	*y* = –0.005*x* ^2^ + 0.159*x*–0.453	0.729	<0.001
HDL‐C/LDL‐C	4.53 ± 0.15^ab^	4.64 ± 0.15^b^	4.23 ± 0.17^ab^	4.22 ± 0.49^ab^	3.95 ± 0.20^ab^	3.58 ± 0.39^a^	*y* = –0.008*x* ^2^ + 0.083*x* + 4.350	0.288	0.028
AST (U/L)	16.35 ± 0.62^a^	17.82 ± 0.15^a^	19.28 ± 0.74^a^	17.92 ± 0.77^a^	23.02 ± 1.37^b^	25.03 ± 1.25^b^	*y* = 0.824*x* + 10.844	0.706	<0.001
ALT (U/L)	9.44 ± 0.95^a^	11.11 ± 0.50^abc^	11.36 ± 0.89^abc^	10.88 ± 0.12^ab^	13.42 ± 0.92^c^	12.66 ± 0.72^bc^	*y* = 0.322*x* + 7.937	0.441	0.003

*Note*: Values are mean ± SEM (*n* = 3 tanks per treatment; one pooled sample per tank). Means within the same row that do not share a common superscript letter are significantly different (*p* < 0.05).

Abbreviations: ALT, alanine aminotransferase; AST, aspartate aminotransferase; HDL‐C, high‐density lipoprotein cholesterol; LDL‐C, low‐density lipoprotein cholesterol; TAG, triglyceride; TC, total cholesterol.

### 3.4. Hepatic Lipid Metabolism–Related Enzyme Activities

Hepatic FAS, MDH, and G6PD activities decreased as dietary lipid increased to 12% but increased at 14%–16% lipid (Table 6). Hepatic LPL activity was significantly lower in the 6% lipid group than in all other groups (*p* < 0.05). Hepatic HSL activity was significantly lower in fish fed 6%–12% lipid than in those fed 14% or 16% lipid. In contrast, hepatic CPT‐1 activity increased with dietary lipid level; however, differences among the 6%–12% lipid groups were not significant (*p* > 0.05).

**Table 6:** Effect of dietary lipid level on the activities of lipid metabolic enzymes in liver of leopard coral grouper (*Plectropomus leopardus*).

Parameter	Dietary lipid levels (%)						Regression analysis		
	6	8	10	12	14	16	Regression equation	*R* ^2^	*p*‐Value
FAS (U/mg protein)	0.46 ± 0.04^c^	0.40 ± 0.00^ab^	0.40 ± 0.01^ab^	0.38 ± 0.01^a^	0.41 ± 0.00^ab^	0.44 ± 0.02^bc^	*y* = 0.003*x* ^2^–0.057*x* + 0.705	0.368	0.002
MDH (U/g protein)	14.57 ± 0.77^c^	12.76 ± 0.38^ab^	12.01 ± 0.44^a^	11.51 ± 0.66^a^	14.10 ± 0.23^bc^	13.82 ± 0.48^bc^	*y* = 0.095*x* ^2^–2.092*x* + 23.50	0.363	0.001
G6PD (U/g protein)	100.77 ± 8.55^d^	92.21 ± 2.05^bc^	86.54 ± 2.15^b^	73.93 ± 2.05^a^	93.87 ± 0.70^bcd^	98.76 ± 1.26^cd^	*y* = 0.794*x* ^2^–17.879*x* + 182.35	0.528	<0.001
LPL (U/mg protein)	0.10 ± 0.00^a^	0.12 ± 0.00^b^	0.12 ± 0.00^b^	0.12 ± 0.00^b^	0.12 ± 0.00^b^	0.13 ± 0.00^b^	*y* = –0.002*x* ^2^ + 0.00565*x* + 0.077	0.351	0.009
HSL (U/mg protein)	0.50 ± 0.01^a^	0.53 ± 0.01^a^	0.53 ± 0.01^a^	0.52 ± 0.00^a^	0.58 ± 0.00^b^	0.58 ± 0.00^b^	*y* = 0.0004*x* ^2^–0.0016*x* + 0.501	0.708	<0.001
CPT‐1 (U/g protein)	0.16 ± 0.00^a^	0.18 ± 0.00^ab^	0.18 ± 0.01^ab^	0.17 ± 0.00^ab^	0.19 ± 0.00^b^	0.21 ± 0.00^c^	*y* = 0.004*x* + 0.133	0.569	<0.001

*Note*: Values are mean ± SEM (*n* = 3 tanks per treatment; one pooled sample per tank). Means within the same row that do not share a common superscript letter are significantly different (*p* < 0.05).

Abbreviations: CPT‐1, carnitine palmityltransferase‐1; FAS, fatty acid synthase; G6PD, glucose‐6‐phosphate dehydrogenase; HSL, hormone‐sensitive lipase; LPL, lipoprotein lipase; MDH, malate dehydrogenase

### 3.5. Liver Histology

**Figure 2:**
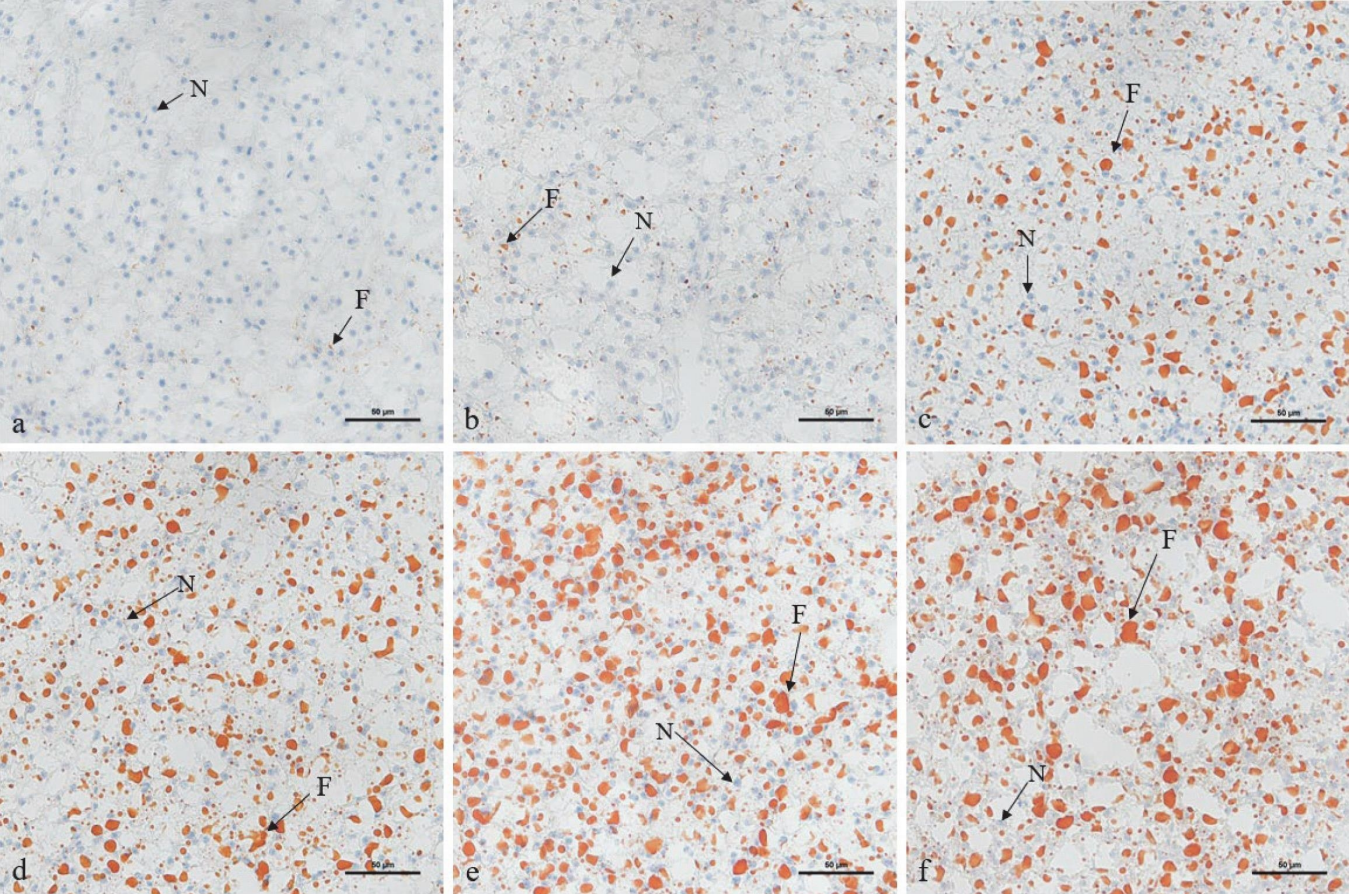
Effect of dietary lipid level on hepatic lipid deposition in leopard coral grouper (*Plectropomus leopardus*), as shown by Oil Red O staining (400×; scale bar = 50 μm). (a) 6% lipid group. (b) 8% lipid group. (c) 10% lipid group. (d) 12% lipid group. (e) 14% lipid group. (f) 16% lipid group. N, nucleus; F, lipid droplets.

**Figure 3:**
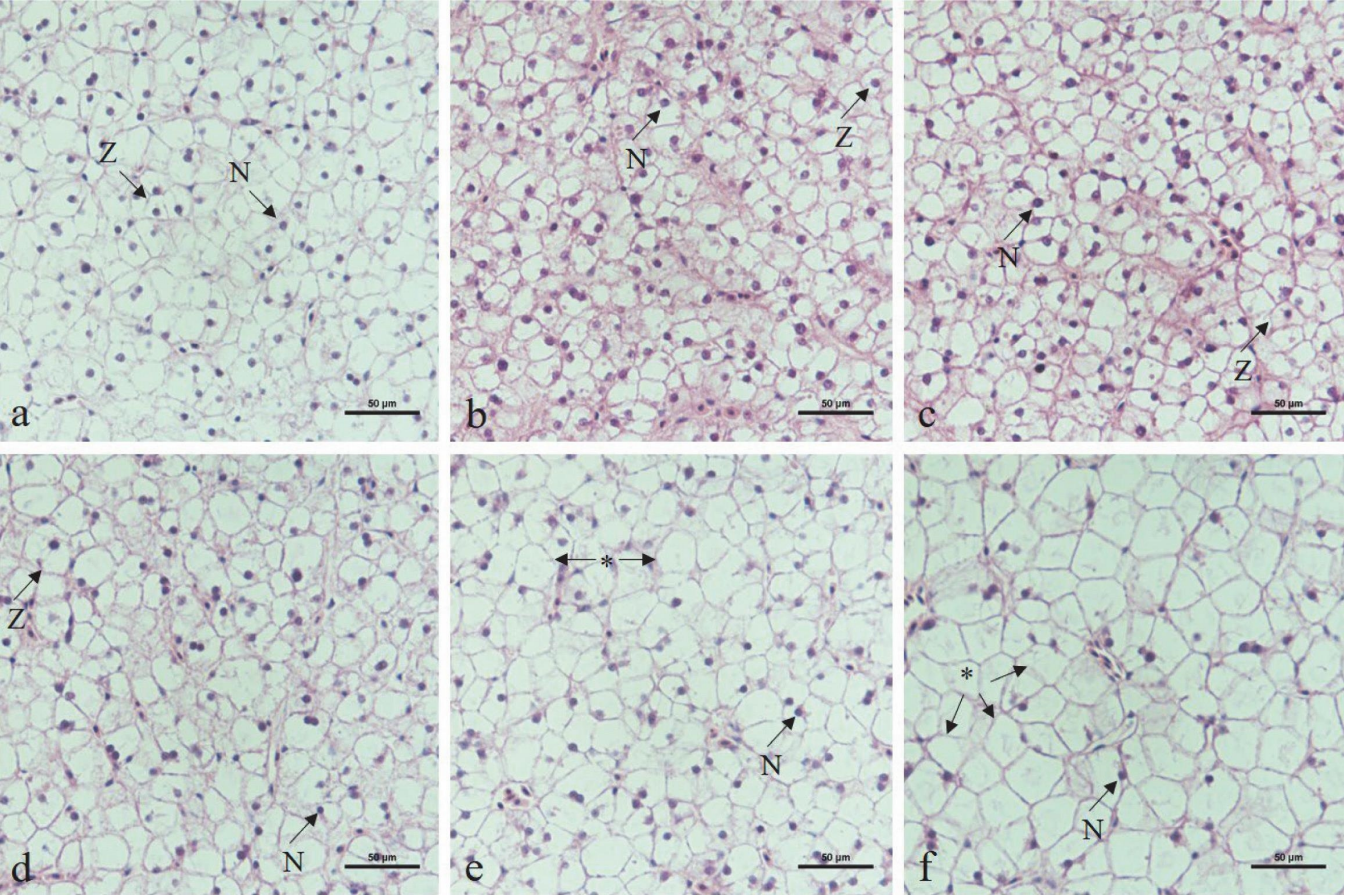
Effects of dietary lipid level on hepatic histomorphology in leopard coral grouper (*Plectropomus leopardus*) (H&E staining). Photomicrographs (400×; scale bar = 50 μm): (a) 6% lipid group, (b) 8% lipid group, (c) 10% lipid group, (d) 12% lipid group, (e) 14% lipid group, and (f) 16% lipid group. N, nucleus; Z, normal hepatocyte with a centrally located nucleus;  ^∗^, nuclear atrophy or disappearance induced by lipid deposition.

Oil Red O staining showed a progressive increase in the size and number of hepatic lipid vacuoles as dietary lipid level increased (Figure 2). Lipid vacuoles were sparse at 6%–8% lipid, became evident at 10%–12% lipid, and were abundant at 14%–16% lipid. Semiquantitative analysis confirmed these findings, showing that lipid vacuole area increased significantly with dietary lipid, reaching the highest values at 14%–16% (Figure S1). In fish fed 6%–12% lipid, hepatocytes exhibited normal morphology with centrally located, round nuclei (Figure 3). In contrast, hepatocytes in the 14%–16% lipid groups showed extensive vacuolization, and most cells displayed nuclear atrophy or loss.

### 3.6. Proximate Composition of Whole Body, Dorsal Muscle, and Liver

Whole‐body moisture decreased as dietary lipid increased up to 10% and then remained stable; moisture was significantly higher in fish fed 6% lipid than in those fed 10%–16% lipid (*p* < 0.05; Table 7). Conversely, whole‐body crude lipid increased with dietary lipid up to 10% and then remained stable at higher lipid levels. Whole‐body ash and crude protein did not differ among dietary treatments (*p* > 0.05).

**Table 7:** Effect of dietary lipid level on the whole body, dorsal muscle, and liver composition in leopard coral grouper (wet weight basis, %).

Parameter	Dietary lipid levels (%)						Regression analysis		
	6	8	10	12	14	16	Regression equation	*R* ^2^	*p*‐Value
Whole body									
Moisture	73.08 ± 0.39^b^	72.39 ± 0.36^ab^	71.54 ± 0.37^a^	71.64 ± 0.27^a^	71.32 ± 0.58^a^	71.29 ± 0.19^a^	*y* = 0.026*x* ^2^–0.744*x* + 76.600	0.638	0.002
Crude lipid	2.37 ± 0.01^a^	3.08 ± 0.05^b^	3.85 ± 0.14^c^	3.73 ± 0.00^c^	3.58 ± 0.18^c^	3.86 ± 0.16^c^	*y* = –0.026*x* ^2^ + 0.698*x*–0.817	0.810	<0.001
Crude protein	19.47 ± 0.26	19.49 ± 0.32	19.02 ± 0.15	18.94 ± 0.31	18.80 ± 0.87	18.93 ± 0.52	—	—	—
Ash	4.95 ± 0.05	4.68 ± 0.05	4.86 ± 0.1	4.90 ± 0.03	5.03 ± 0.25	4.93 ± 0.11	—	—	—
Dorsal muscle									
Moisture	77.93 ± 0.18	78.56 ± 0.47	78.04 ± 0.26	77.93 ± 0.09	77.85 ± 0.04	77.97 ± 0.31	—	—	—
Crude lipid	0.64 ± 0.02^a^	0.70 ± 0.01^a^	0.67 ± 0.06^a^	1.02 ± 0.06^b^	1.04 ± 0.07^b^	0.99 ± 0.04^b^	*y* = –0.002*x* ^2^ + 0.078*x* + 0.188	0.653	<0.001
Crude protein	20.80 ± 0.13	20.41 ± 0.33	20.44 ± 0.06	20.90 ± 0.05	20.82 ± 0.09	20.83 ± 0.21	—	—	—
Liver									
Moisture	67.76 ± 0.76^b^	67.63 ± 0.71^b^	67.24 ± 0.55^b^	64.85 ± 0.04^a^	64.91 ± 0.40^a^	62.99 ± 1.17^a^	*y* = –0.033*x* ^2^ + 0.229*x* + 37.700	0.732	<0.001
Crude lipid	6.50 ± 0.08^a^	6.46 ± 0.25^a^	8.96 ± 0.69^ab^	9.01 ± 0.75^ab^	10.44 ± 0.81^b^	12.00 ± 0.74^c^	*y* = –0.582*x* + 2.698	0.841	<0.001
Crude protein	11.49 ± 0.16^b^	10.43 ± 0.48^b^	9.81 ± 0.38^a^	9.87 ± 0.06^a^	9.76 ± 0.35^a^	10.06 ± 0.03^a^	*y* = 0.039*x* ^2^–0.993*x* + 15.953	0.648	<0.001

*Note*: Values are mean ± SEM (*n* = 3 tanks per treatment; one pooled sample per tank). Means within the same row that do not share a common superscript letter are significantly different (*p*  < 0.05).

**Figure 4:**
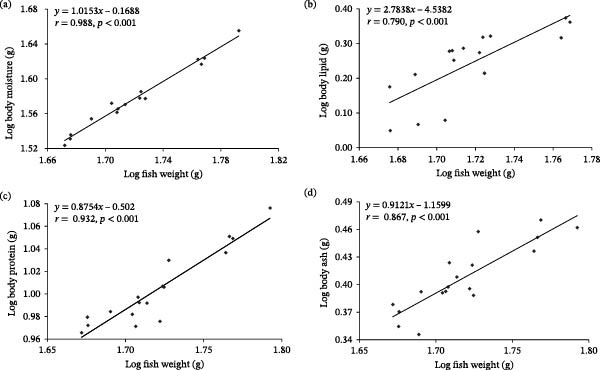
Allometric relationships between log_10_‐transformed whole‐body composition variables and log_10_‐transformed body weight in leopard coral grouper (*Plectropomus leopardus*) fed experimental diets with varying lipid levels: (a) moisture, (b) lipid, (c) protein, and (d) ash.

Allometric analysis indicated that whole‐body crude protein (*b* = 0.8754; Figure 4c) and ash (*b* = 0.9121; Figure 4d) increased less than proportionally with body weight (*b* < 1), whereas crude lipid increased more than proportionally (*b* = 2.7838; Figure 4b). Whole‐body moisture showed an approximately proportional relationship with body weight (*b* ≈ 1; Figure 4a).

Dorsal muscle crude lipid was significantly higher in fish fed 12%–16% lipid than in those fed 6%–10% lipid (*p* < 0.05; Table 7), whereas dorsal muscle moisture and crude protein did not differ among dietary treatments (*p* > 0.05).

Hepatic crude protein and moisture decreased as dietary lipid increased to 10% and 12%, respectively, and then remained stable (Table 7). Hepatic moisture was higher in fish fed 6%–10% lipid than in those fed 12%–16% lipid, and hepatic crude protein was significantly higher in the 6% and 8% groups than in all other groups (*p* < 0.05). In contrast, hepatic crude lipid increased linearly with dietary lipid level.

### 3.7. Fatty Acid Profile of Dorsal Muscle

As dietary lipid level increased, dorsal muscle monounsaturated fatty acids (MUFAs), C18:2n‐6 (linoleic acid), n‐6 polyunsaturated fatty acids (PUFAs), C18:3n‐3 (α‐linolenic acid), and C20:5n‐3 (eicosapentaenoic acid [EPA]) increased linearly (Table 8). In contrast, the proportions of saturated fatty acids (SFAs), C22:6n‐3 (docosahexaenoic acid [DHA]), and n‐3 PUFA decreased linearly, as did the n‐3/n‐6 PUFA and DHA/EPA ratios.

**Table 8:** Effect of dietary lipid level on fatty acid composition (% of total fatty acids) in dorsal muscle of leopard coral grouper (*Plectropomus leopardus*).

Fatty acids	Dietary lipid levels (%)						Regression analysis		
	6	8	10	12	14	16	Regression equation	*R* ^2^	*p*‐Value
C14 : 0	3.79 ± 0.14^d^	2.67 ± 0.08^c^	2.23 ± 0.04^b^	1.81 ± 0.13^a^	1.69 ± 0.06^a^	1.53 ± 0.05^a^	*y* = –0.210*x* + 4.590	0.839	<0.001
C16 : 0	24.86 ± 0.20^e^	22.12 ± 0.26^d^	20.50 ± 0.13^c^	19.26 ± 0.26^b^	17.77 ± 0.03^a^	17.41 ± 0.38^a^	*y* = –0.736*x* + 28.415	0.933	<0.001
C18 : 0	7.57 ± 0.00^d^	7.21 ± 0.04^c^	7.16 ± 0.13^c^	6.45 ± 0.09^b^	5.95 ± 0.10^a^	5.70 ± 0.16^a^	*y* = –0.198*x* + 8.844	0.920	<0.001
C20 : 0	0.36 ± 0.00^a^	0.40 ± 0.01^b^	0.41 ± 0.01^b^	0.44 ± 0.00^c^	0.45 ± 0.00^c^	0.44 ± 0.01^c^	*y* = 0.008*x* + 0.323	0.810	<0.001
∑SFA	36.57 ± 0.06^e^	32.39 ± 0.27^d^	30.30 ± 0.25^c^	27.96 ± 0.24^b^	25.85 ± 0.14^a^	25.08 ± 0.44^a^	*y* = 1.135*x* + 42.173	0.950	<0.001
C16 : 1	3.16 ± 0.09^d^	2.58 ± 0.03^c^	2.32 ± 0.08^b^	2.29 ± 0.02^b^	2.20 ± 0.03^ab^	2.09 ± 0.08^a^	*y* = –0.093*x* + 3.468	0.758	<0.001
C18 : 1	20.13 ± 0.18^a^	21.63 ± 0.27^b^	22.32 ± 0.17^c^	23.14 ± 0.26^d^	24.25 ± 0.07^e^	24.67 ± 0.07^e^	*y* = 0.448*x* + 17.758	0.951	<0.001
C20 : 1	1.99 ± 0.00	1.98 ± 0.05	1.95 ± 0.01	1.95 ± 0.02	1.96 ± 0.01	1.94 ± 0.02	—	—	—
∑MUFA	25.28 ± 0.27^a^	26.19 ± 0.29^b^	26.59 ± 0.23^b^	27.38 ± 0.29^c^	28.41 ± 0.08^d^	28.70 ± 0.13^d^	*y* = 0.351*x* + 23.233	0.917	<0.001
C18 : 2n‐6	14.75 ± 0.12^a^	18.26 ± 0.58^b^	20.39 ± 0.34^c^	21.76 ± 0.89^c^	24.70 ± 0.10^d^	25.62 ± 0.39^d^	*y* = 1.072*x* + 9.121	0.942	<0.001
C20 : 2n‐6	0.75 ± 0.01^c^	0.74 ± 0.01^c^	0.68 ± 0.01^b^	0.64 ± 0.02^ab^	0.63 ± 0.02^a^	0.61 ± 0.02^a^	*y* = –0.016*x* + 0.849	0.828	<0.001
C20 : 4n‐6	0.80 ± 0.01	0.88 ± 0.07	0.87 ± 0.02	0.89 ± 0.07	0.78 ± 0.00	0.83 ± 0.07	—	—	—
∑n‐6 PUFA	16.30 ± 0.11^a^	19.87 ± 0.50^b^	21.94 ± 0.32^c^	23.29 ± 1.39^d^	26.11 ± 0.11^e^	26.98 ± 0.36^e^	*y* = 1.054*x* + 10.831	0.946	<0.001
C18 : 3n‐3	1.53 ± 0.02^a^	1.96 ± 0.01^b^	2.19 ± 0.06^c^	2.51 ± 0.04^d^	2.78 ± 0.02^e^	2.94 ± 0.05^f^	*y* = 0.141*x* + 0.771	0.970	<0.001
C20 : 5n‐3	4.97 ± 0.05^a^	4.90 ± 0.07^a^	4.95 ± 0.05^a^	5.23 ± 0.03^b^	5.22 ± 0.05^b^	5.34 ± 0.06^b^	*y* = 0.044*x* + 4.616	0.672	<0.001
C22 : 6n‐3	10.01 ± 0.28^c^	9.48 ± 0.03^c^	9.48 ± 0.20^c^	8.62 ± 0.17^b^	7.83 ± 0.13^a^	7.54 ± 0.18^a^	*y* = –0.259*x* + 11.677	0.882	<0.001
∑n‐3 PUFA	16.50 ± 0.25^b^	16.34 ± 0.08^b^	16.61 ± 0.12^b^	16.36 ± 0.10^b^	15.83 ± 0.17^a^	15.81 ± 0.06^a^	*y* = –0.075*x* + 17.065	0.468	0.002
n‐3/n‐6 PUFA	1.01 ± 0.02^e^	0.82 ± 0.02^d^	0.76 ± 0.01^c^	0.68 ± 0.01^b^	0.61 ± 0.01^a^	0.58 ± 0.01^a^	*y* = –0.041*x* + 1.196	0.902	<0.001
DHA/EPA	2.01 ± 0.06^c^	1.94 ± 0.02^c^	1.92 ± 0.06^c^	1.65 ± 0.05^b^	1.50 ± 0.01^a^	1.41 ± 0.05^a^	*y* = –0.065*x* + 2.455	0.876	<0.001

*Note:* Values are mean ± SEM (*n* = 3 tanks per treatment; one pooled sample per tank). Means within the same row that do not share a common superscript letter are significantly different (*p*  < 0.05). DHA/EPA: 22:6n‐3/20:5n‐3.

Abbreviations: MUFA, monounsaturated fatty acids; n‐3 PUFA, n‐3 polyunsaturated fatty acids; n‐6 PUFA, n‐6 polyunsaturated fatty acids; SFA, saturated fatty acids.

### 3.8. Antioxidant‐Related Parameters

Serum SOD activity increased with dietary lipid up to 12% and declined at higher lipid levels (Table 9). Similarly, serum GPx and TAC increased up to 8% and 10% lipid, respectively, and decreased thereafter. Serum MDA increased with dietary lipid level; however, differences among the 6%–12% lipid groups were not significant (*p* > 0.05).

**Table 9:** Effect of dietary lipid level on antioxidant‐related indices in serum, liver, and intestine of leopard coral grouper (*Plectropomus leopardus*).

Parameter	Dietary lipid level (%)						Regression analysis		
	6	8	10	12	14	16	Regression equation	*R* ^2^	*p*‐Value
Serum									
SOD (U/mL)	27.90 ± 0.85^b^	29.27 ± 1.99^b^	32.05 ± 0.29^bc^	34.83 ± 1.45^c^	30.25 ± 0.37^bc^	21.60 ± 3.12^a^	*y* = –0.367*x* ^2^ + 7.723*x*–6.819	0.628	<0.001
GPx (mmol/L)	1.31 ± 0.03^b^	1.65 ± 0.01^c^	1.74 ± 0.02^c^	1.25 ± 0.18^b^	0.94 ± 0.04^a^	0.85 ± 0.02^a^	*y* = –0.017*x* ^2^ + 0.302*x* + 0.229	0.653	<0.001
TAC (mmol/L)	1.12 ± 0.01^a^	1.16 ± 0.00^b^	1.16 ± 0.00^b^	1.12 ± 0.02^a^	1.12 ± 0.00^a^	1.10 ± 0.01^a^	*y* = –0.002*x* ^2^ + 0.030*x* + 1.008	0.485	0.001
MDA (nmol/mL)	18.77 ± 1.90^a^	16.98 ± 3.90^a^	17.87 ± 2.37^a^	25.47 ± 1.73^ab^	34.31 ± 1.97^b^	50.27 ± 7.21^c^	*y* = 3.038*x*–5.896	0.620	<0.001
Liver									
SOD (U/μg protein)	0.36 ± 0.02^a^	0.38 ± 0.02^a^	0.39 ± 0.02^ab^	0.39 ± 0.01^ab^	0.41 ± 0.02^ab^	0.46 ± 0.03^b^	*y* = 0.008*x* + 0.305	0.322	0.006
GPx (U/mg protein)	0.17 ± 0.00^a^	0.20 ± 0.00^b^	0.22 ± 0.02^b^	0.20 ± 0.00^b^	0.27 ± 0.02^c^	0.26 ± 0.00^c^	*y* = 0.0097*x* + 0.112	0.632	<0.001
TAC (μmol/g protein)	7.66 ± 0.41^a^	9.23 ± 0.36^ab^	11.91 ± 0.64^c^	10.50 ± 0.84^bc^	8.89 ± 0.87^ab^	9.10 ± 0.81^ab^	*y* = –0.108*x* ^2^ + 2.465*x*–3.142	0.369	0.002
MDA (nmol/mg protein)	0.85 ± 0.04^a^	0.87 ± 0.11^a^	0.87 ± 0.06^a^	1.22 ± 0.06^ab^	1.85 ± 0.73^bc^	2.17 ± 0.06^c^	*y* = 0.141*x*–0.277	0.423	0.001
Hindgut									
SOD (U/μg protein)	0.26 ± 0.00^a^	0.30 ± 0.01^b^	0.38 ± 0.00^c^	0.31 ± 0.01^b^	0.30 ± 0.01^b^	0.30 ± 0.00^b^	*y* = –0.003*x* ^2^ + 0.064*x*–0.0189	0.421	0.002
GPx (U/mg protein)	0.12 ± 0.02^a^	0.13 ± 0.01^a^	0.18 ± 0.00^bc^	0.15 ± 0.00^b^	0.19 ± 0.01^c^	0.20 ± 0.00^c^	*y* = 0.008*x* + 0.078	0.563	<0.001
TAC (μmol/g protein)	18.50 ± 0.42^a^	19.13 ± 0.76^ab^	19.42 ± 0.07^ab^	18.53 ± 0.07^a^	21.79 ± 0.30^c^	20.30 ± 0.79^bc^	*y* = 0.0009*x* ^2^ + 0.213*x* + 17.190	0.272	0.004
MDA (nmol/mg protein)	1.79 ± 0.11^a^	1.89 ± 0.18^a^	2.18 ± 0.38^a^	5.86 ± 1.06^b^	5.32 ± 1.70^b^	8.99 ± 0.24^c^	*y* = 0.685*x*–3.340	0.523	<0.001

*Note*: Values are mean ± SEM (*n* = 3 tanks per treatment; one pooled sample per tank). Means within the same row that do not share a common superscript letter are significantly different (*p*  < 0.05).

Abbreviations: GPx, glutathione peroxidase; MDA, malondialdehyde; SOD, superoxide dismutase; TAC, total antioxidant capacity.

Hepatic SOD and GPx increased with dietary lipid level (Table 9). Hepatic MDA also increased overall, although differences among the 6%–12% lipid groups were not significant (*p* > 0.05). Hepatic TAC increased up to 10% lipid and declined at higher lipid levels.

Hindgut SOD increased up to 10% lipid and declined thereafter (Table 9). Hindgut TAC increased up to 14% lipid and declined thereafter. In contrast, hindgut GPx and MDA increased with dietary lipid level; however, MDA did not differ among the 6%–10% lipid groups (*p* > 0.05).

## 4. Discussion

Fish require appropriate dietary lipid levels to support optimal growth and normal physiological development [29, 30]. As an essential nutrient in aquafeeds, lipids provide concentrated energy, spare dietary protein, and supply essential fatty acids. Despite its high economic value, the dietary lipid requirement of *P. leopardus* has not been quantitatively defined. Here, we addressed this gap by estimating the dietary lipid requirement of juvenile *P. leopardus* under the present rearing conditions. Based on ANOVA, growth performance was highest at ~10% dietary lipid under the present experimental conditions. Quadratic regression based on WG suggested that the dietary lipid level was ~9.3% for juveniles (13.94 ± 0.07 g), with a 95% CI of 8.62%–9.98%. However, the relatively low *R* ^2^ value indicates limited model strength, and this estimate should therefore be interpreted with caution. Comparable estimates derived from FBW, SGR, and PER suggest that the growth response to dietary lipid levels was generally consistent across indices and likely falls within a relatively narrow range. Similar values have been reported in other Epinephelinae species, including 8.7% in juvenile Malabar grouper (*Epinephelus malabaricus*; 4.43 ± 0.07 g) [25], 9.1% in redspotted grouper (*Epinephelus akaara*; ≈2.51 g) [30], and 9.3% in kelp grouper (*Epinephelus moara*; 5.87 ± 0.09 g) [26]. Collectively, these studies suggest that dietary lipid levels associated with improved growth are broadly similar across many *Epinephelinae* species (often near 9%) but can vary with species, developmental stage, and diet formulation. For example, a substantially higher optimum (16.0%) has been reported for early juvenile orange‐spotted grouper (*E. coioides*; 0.071 ± 0.002 g) [31]. The growth enhancement at 9.3% lipid likely reflects an adequate energy supply and a protein‐sparing effect, as indicated by the concurrent increase in PER, consistent with reports in grouper species (e.g., kelp grouper *E. moara*) [26]. In contrast, excessive dietary lipid (12%–16%) increased dietary energy density and depressed FI while promoting visceral and hepatic lipid deposition (higher VSI and HSI) and thereby limiting nutrient partitioning toward somatic growth. These responses may involve energy‐sensing and lipid‐regulatory pathways (e.g., AMPK and PPAR/SREBP signaling), although confirmation at the transcriptional level is warranted [32, 33].

Digestive enzyme activities are widely used as functional indicators of digestive capacity and nutritional adaptation to dietary macronutrient composition in fish [34]. Consistent with previous studies [31], intestinal lipase activity increased linearly with dietary lipid level, suggesting an adaptive, substrate‐driven enhancement of lipid digestion, potentially mediated by increased bile secretion and/or intestinal lipase secretion [17, 35]. In contrast, amylase activity decreased as dietary lipid increased, consistent with reports in *Sparus aurata* [36], which may reflect a shift in energy utilization toward lipid oxidation and a reduced demand for carbohydrate digestion. Trypsin activity was unaffected, likely due to the similar dietary protein levels among diets. Together, these patterns indicate a shift in digestive capacity toward lipid utilization as dietary lipid increases.

Serum lipid indices (HDL‐C, LDL‐C, TC, and TAG) are commonly used indicators of lipid absorption and transport and of cholesterol homeostasis in fish [37]. Consistent with reports in large yellow croaker [18], largemouth bass [20], and kelp grouper [26], increasing dietary lipid elevated serum TC, TAG, HDL‐C, and LDL‐C in the present study, suggesting enhanced lipid absorption and transport in *P. leopardus*. The concurrent increases in HDL‐C and LDL‐C suggest that both reverse cholesterol transport and peripheral lipid/cholesterol delivery were upregulated in response to the higher lipid load, potentially through increased lipoprotein assembly and circulation [38]. Notably, the HDL‐C/LDL‐C ratio remained relatively stable, suggesting coordinated cholesterol transport and indicating that systemic lipid homeostasis was largely maintained within the tested range, even at higher lipid levels.

The liver is a central organ governing lipid metabolism in fish [39]. Accumulating evidence indicates that optimal dietary lipid levels tend to suppress lipogenic pathways (e.g., FAS‐related activity/expression) while promoting lipid catabolic/oxidative pathways (e.g., CPT‐1–mediated β‐oxidation) [15, 20]. Consistent with this concept, dietary lipid up to 12% suppressed lipogenic enzyme activities (FAS, MDH, and G6PD), whereas 14%–16% lipid increased these activities and was accompanied by higher activities of lipid catabolic/oxidative enzymes (HSL, LPL, and CPT‐1). These results suggest a biphasic hepatic metabolic response to increasing dietary lipid. At moderate lipid inclusion (8%–12%), lower activities of lipogenic enzymes suggest reduced NADPH availability and diminished de novo lipogenesis [40], whereas the increase in CPT‐1 indicates an enhanced capacity for mitochondrial fatty acid entry and β‐oxidation [33]. This coordinated adjustment may improve energy utilization efficiency and thereby support growth. However, at 14%–16% lipid, lipid influx likely exceeded the liver’s oxidative and export capacity, as supported by marked lipid droplet accumulation (Oil Red O staining), hepatocyte vacuolization with nuclear atrophy or loss, elevated hepatic lipid content, and increased serum transaminases [41]. Although lipid catabolism/oxidation (e.g., HSL and CPT‐1) appeared to be upregulated, this compensatory response was insufficient, resulting in excessive hepatic lipid deposition and impaired liver function. This imbalance may partly explain the growth depression observed in the high‐lipid groups. Similar biphasic responses have been reported in largemouth bass and other species fed high‐lipid diets [20]. Likewise, high dietary lipid–induced hepatic lipid deposition has been negatively associated with growth performance in other grouper species [26, 27]. These shifts may involve altered regulation of lipid metabolism (e.g., PPAR‐ and SREBP‐associated signaling) and warrant further validation at the molecular level [33].

Oxidative stress is a major concern in fish fed high‐fat diets [16]. In the present study, moderate dietary lipid levels (8%–12%) enhanced antioxidant capacity, as reflected by increased SOD, GPx, and TAC in serum, liver, and hindgut. In contrast, MDA increased markedly at 14%–16% lipid, indicating enhanced lipid peroxidation and elevated oxidative stress. Similar patterns have been reported in largemouth bass [20]. Excessive dietary lipid likely increases ROS production via enhanced β‐oxidation and mitochondrial respiration, thereby promoting PUFA peroxidation and amplifying lipid radical chain reactions [20]. Under these conditions, antioxidant defenses may be insufficient to counteract ROS generation, resulting in oxidative damage that compromises hepatocellular integrity and may contribute to the elevated serum transaminases observed in the high‐lipid groups. Collectively, these findings suggest that moderate lipid inclusion can induce an adaptive antioxidant response, whereas excessive lipid intake promotes lipid peroxidation; this oxidative burden could contribute to hepatocellular impairment and help explain the elevated transaminases and reduced growth observed in high‐lipid treatments.

Dietary lipid level also influences tissue fatty acid composition in fish. Consistent with previous reports [31, 42, 43], the dorsal muscle fatty acid profile largely mirrored the dietary fatty acid composition, indicating substantial dietary deposition of fatty acids in muscle. Increasing dietary lipid significantly reduced the n‐3/n‐6 PUFA ratio in the dorsal muscle. This reduction may reflect greater incorporation of C18 : 2n‐6 (from soybean oil) into neutral lipid stores as tissue lipid deposition increases, which dilutes the relative proportion of long‐chain n‐3 PUFA. Because n‐3 PUFAs contribute to sensory quality and human health benefits, a lower n‐3/n‐6 ratio may reduce fillet nutritional value [44, 45]. For this premium species, these results highlight a performance–quality trade‐off and suggest that dietary lipid level should be optimized together with lipid source to maintain desirable fillet n‐3/n‐6 characteristics. The consistently higher DHA than EPA in muscle likely reflects selective retention of DHA and/or preferential utilization of EPA as an oxidative substrate because DHA is often conserved for structural roles in membranes [14, 31, 46]. Although the DHA/EPA ratio declined as dietary lipid increased, it remained >1.0 across treatments, indicating a DHA‐rich fillet fatty acid profile with potential neuroprotective and cardioprotective benefits [47].

Consistent with previous research [46, 48–50], whole‐body and muscle lipid contents increased with dietary lipid level. Based on Shearer’s allometric model [51], carcass lipid increased more than proportionally with body weight (*b* = 2.7838), indicating a strong capacity for lipid deposition in *P. leopardus*. Although dietary lipid level clearly influenced whole‐body proximate composition, further work is needed to disentangle the effects of diet from endogenous factors (e.g., body size and developmental stage) on body composition.

This study addresses the lack of quantitative information on the dietary lipid requirement of juvenile *P. leopardus* by estimating an optimal lipid level of 9.3% based on quadratic regression of WG, using isonitrogenous diets (~53% crude protein) with a 1:1 fish oil:soybean oil blend. Considering both growth and health‐related responses, a dietary lipid range of 8%–12% appears appropriate for juveniles (~14 g). In contrast, lipid levels ≥14% should be used cautiously because they were associated with higher HSI/VSI and hepatic lipid accumulation, increased lipid peroxidation, and elevated serum transaminases, collectively indicating hepatic metabolic overload that may constrain growth under excessive lipid intake.

## 5. Conclusion

Dietary lipid levels exhibited a dose‐dependent effect on growth performance in juvenile *P. leopardus*, with growth increasing as lipid levels rose from 6% to ~10% and declining at higher levels. Moderate lipid inclusion (8%–12%) supported improved growth and metabolic balance, whereas excessive lipid levels (≥14%) led to hepatic lipid accumulation, increased oxidative stress, and impaired growth. Regression analysis suggested an approximate optimum near 9.3%; however, this value should be interpreted with caution due to limited model strength. These results provide a practical reference for formulated feed development and precision nutrition in *P. leopardus* aquaculture.

## Author Contributions

**Xiangqin Lin and Yixiong Cao**: writing – original draft, visualization, validation, methodology. **Xiaoxue Meng, Shiwei Xie, Shuyan Chi, Shuang Zhang, and Beiping Tan**: visualization, validation, methodology, data curation, conceptualization, formal analysis. **Junming Deng**: writing – review and editing, supervision, resources, funding acquisition, project administration.

## Funding

This work was supported by the China Agriculture Research System (CARS‐47) and the Program for Scientific Research Startup Funds of Guangdong Ocean University (Grant 060302022007).

## Disclosure

All authors have approved the submitted version.

## Conflicts of Interest

The authors declare no conflicts of interest.

## Supporting Information

Additional supporting information can be found online in the Supporting Information section.

## Supporting information


**Supporting Information** Figure S1: Effect of dietary lipid level on hepatic lipid vacuole area (%) in leopard coral grouper (*Plectropomus leopardus*). Values are means with their standard errors represented by vertical bars (*n* = 3). Different lowercase letters above the bars indicate significant differences (*p* < 0.05).

## Data Availability

The data will be made available upon request.
