# Supplementary material for: Optimal Dietary Lipid Requirement for Juvenile Leopard Coral Grouper (Plectropomus leopardus)
Source: Aquac Nutr. 2026 May 4;2026:9438737. doi: 10.1155/anu/9438737 (PMC13137126; doi:10.1155/anu/9438737)
Supplement: Supplementary file 1 — Supporting Information Figure S1: Effect of dietary lipid level on hepatic lipid vacuole area (%) in leopard coral grouper (Plectropomus leopardus). Values are means with their standard errors represented by vertical bars (n = 3). Different lowercase letters above the bars indicate significant differences (p < 0.05). [file ANU-2026-9438737-s001.docx]

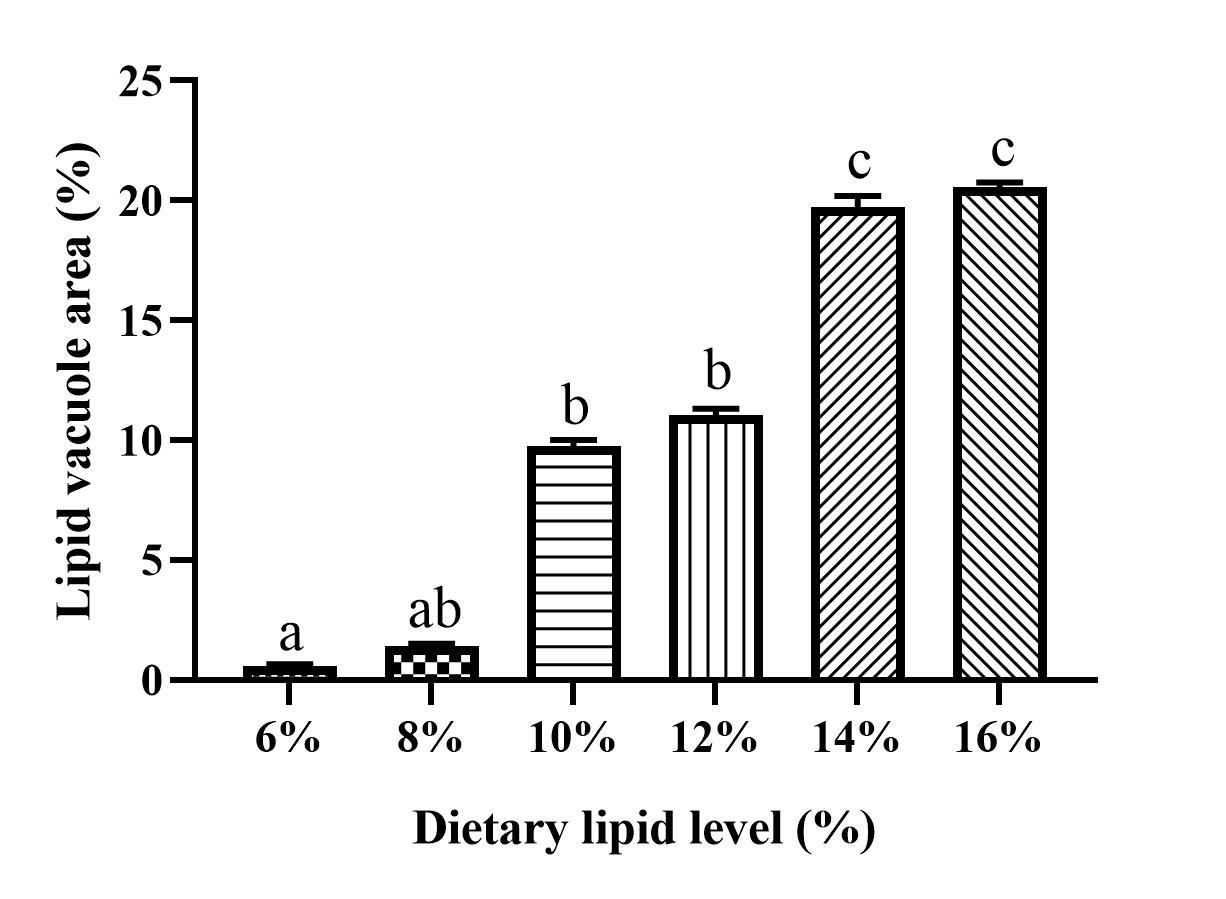


Figure s1: Effect of dietary lipid level on hepatic lipid vacuole area (%) in leopard coral grouper (*Plectropomus leopardus*). Values are means with their standard errors represented by vertical bars (n = 3). Different lowercase letters above the bars indicate significant differences (*P* < 0.05).
